# Transcriptional Alterations of Mouse Trigeminal Ganglion Neurons Following Orofacial Inflammation Revealed by Single-Cell Analysis

**DOI:** 10.3389/fncel.2022.885569

**Published:** 2022-06-02

**Authors:** Qing Liu, Lijia Mai, Shengyan Yang, Shilin Jia, Yanhao Chu, Hongwen He, Wenguo Fan, Fang Huang

**Affiliations:** ^1^Hospital of Stomatology, Guanghua School of Stomatology, Sun Yat-sen University, Guangzhou, China; ^2^Guangdong Provincial Key Laboratory of Stomatology, Guangzhou, China

**Keywords:** primary sensory neuron, trigeminal ganglion, orofacial pain, transcriptome profiling, single-cell RNA-sequencing, complete Freund’s adjuvant

## Abstract

Orofacial inflammation leads to transcriptional alterations in trigeminal ganglion (TG) neurons. However, diverse alterations and regulatory mechanisms following orofacial inflammatory pain in different types of TG neurons remain unclear. Here, orofacial inflammation was induced by injection of complete Freund’s adjuvant (CFA) in mice. After 7 days, we performed single-cell RNA-sequencing on TG cells of mice from control and treatment groups. We identified primary sensory neurons, Schwann cells, satellite glial cells, oligodendrocyte-like cells, immune cells, fibroblasts, and endothelial cells in TG tissue. After principal component analysis and hierarchical clustering, we identified six TG neuronal subpopulations: peptidergic nociceptors (PEP1 and PEP2), non-peptidergic nociceptors (NP1 and NP2), C-fiber low-threshold mechanoreceptors (cLTMR) and myelinated neurons (*Nefh*-positive neurons, NF) based on annotated marker gene expression. We also performed differential gene expression analysis among TG neuronal subtypes, identifying several differential genes involved in the inflammatory response, neuronal excitability, neuroprotection, and metabolic processes. Notably, we identified several potential novel targets associated with pain modulation, including *Arl6ip1*, *Gsk3b*, *Scn7a*, and *Zbtb20* in PEP1, *Rgs7bp* in PEP2, and *Bhlha9* in cLTMR. The established protein–protein interaction network identified some hub genes, implying their critical involvement in regulating orofacial inflammatory pain. Our study revealed the heterogeneity of TG neurons and their diverse neuronal transcriptomic responses to orofacial inflammation, providing a basis for the development of therapeutic strategies for orofacial inflammatory pain.

## Introduction

Sensory neurons located in the trigeminal ganglion (TG) and dorsal root ganglion (DRG) detect and relay mechanical, thermal, and chemical stimuli from the head region and the rest of the body, respectively (Woolf and Ma, 2007; Dubin and Patapoutian, 2010). Neurons are generally classified based on their anatomical, genetic, physiological, and biochemical characteristics (Basbaum et al., 2009; Li et al., 2016). Furthermore, certain neurons are crucial for mediating different sensory modalities, such as touch, temperature, proprioception, and nociception (Chiu et al., 2014; Madden et al., 2020). Nevertheless, the functions and mechanisms of different sensory neurons in various pathological conditions remain largely undetermined.

Orofacial pain is a highly debilitating pain condition that is considered a serious public health problem (Banigo et al., 2018). Alterations in plasticity of primary sensory neurons contribute to the pathogenesis of pain (Woolf and Salter, 2000). Changes in a multitude of genes within TG neurons have been observed in orofacial pain models (Aczel et al., 2018, 2020). Previous transcriptomic studies on orofacial pain have analyzed RNA isolated from bulk TG tissue, which is a mixture of neuronal and non-neuronal cells (Chung et al., 2016; Aczel et al., 2018). However, bulk TG tissue analysis cannot distinguish the various cell types in which these alterations occur and whether these alterations are similar or distinct among different neuronal subtypes. Identifying the detailed transcriptional alterations of different TG neuronal types may reveal potential new targets for pain control.

Single-cell RNA-sequencing (scRNA-seq) is the leading technique used to characterize the transcriptomic profiles of individual cells in heterogeneous samples (Poulin et al., 2016). Migraine-associated gene expression in TG has been studied in detail using scRNA-seq (Vgontzas and Renthal, 2020). However, the overall transcriptomic profiles within TG neuronal subtypes following orofacial inflammation are yet to be characterized at the single-cell level.

In this study, we optimized complete Freund’s adjuvant (CFA) to establish an orofacial inflammatory pain model. After scRNA-seq analysis, we explored the classification of primary sensory neurons in TG, and further bioinformatic analysis elucidated the transcriptomes of distinct neuronal subpopulations upon orofacial inflammatory pain. We unbiasedly classified six subpopulations of TG sensory neurons based on transcriptional patterns and further analysis revealed several differentially expressed genes (DEGs) that were likely to functionally related to pain modulation, such as *Arl6ip1*, *Gsk3b*, *Scn7a*, and *Zbtb20* in peptidergic nociceptor 1 (PEP1), *Rgs7bp* in PEP2, and *Bhlha9* in C-fiber low-threshold mechanoreceptors (cLTMR), supporting the development of novel therapeutics for orofacial inflammatory pain.

## Materials and Methods

### Animals

Adult male C57BL/6 mice (5–8 weeks, 20–25 g) were obtained from the Animal Care Committee for the Care and Use of Laboratory Animals of Sun Yat-sen University (Guangzhou, Guangdong Province, China), and housed under a 12-h light-dark cycle. Food and water could be accessed *ad libitum*. All experimental protocols were approved by the Ethics Committee of the Sun Yat-sen University in China (No. SYSU-IACUC-2020-000245). Twelve animals were divided into control and experimental groups (*n* = 6 each). Orofacial inflammation in mice was induced by injecting of 20 μL CFA (Sigma-Aldrich, St. Louis, MO, United States; 1:1 in 0.9% sterile saline) into the right vibrissal pad. The right TG tissue and spinal trigeminal nucleus (STN) tissues were extracted 7 days after CFA injection. The control group received the same experimental treatment, but CFA was substituted for saline.

### Orofacial Pain Sensitivity Tested With Von Frey Filaments

The mechanical pain thresholds of the mouse whisker pad were measured by von Frey filaments (Touch Test Sensory Evaluators, North Coast Medical, Morgan Hill, CA, United States) in the daytime before and after CFA injection every day. Before experiments, mice were acclimated in the insulating cotton gloves several times until they were calm. The von Frey filaments with increased forces were pressed vertically on mouse right whisker pad surface for about 5 s and each fiber was tested five times at intervals of few minutes. The lowest force of the filaments that produced withdrawal responses (face stroking with the forepaw or head shaking) at least three times was considered as the mechanonociceptive threshold.

### Immunofluorescent Staining

After deeply anesthetized with 1% pentobarbital sodium (50 mg/kg), the mice were perfused transcardially with PBS followed by 4% paraformaldehyde. The right STN tissues were extracted and further fixed using 4% paraformaldehyde at 4°C for 12 h. Then the tissues were cryoprotected with 30% sucrose and embedded in OCT compound. The STN slices (30 μm) were permeabilized with 0.1% Triton X-100 (Sigma-Aldrich, St. Louis, MO, United States) and blocked with 5% bovine serum albumin (BSA, BioFroxx, Germany) for 1 h at room temperature. The sections were incubated for 24 h at 4°C with rabbit anti-c-Fos antibody (1:500, No. 2250, Cell Signaling Technology, Danvers, MA, United States). After washing with PBS three times for 15 min, the slices were incubated with secondary DyLight^®^ 594 donkey anti-rabbit IgG (1:400, EarthOx, Burlingame, CA, United States) for 1 h at room temperature. Images were captured using LSM780 laser confocal scanning microscope (Zeiss, Oberkochen, Germany). The mean fluorescence intensity was calculated using ImageJ software for quantitative analysis of the expression level of c-Fos in STN neurons.

### Single-Cell Suspension Preparation

Mice were anesthetized with 4% isoflurane inhalation and sacrificed by decapitation, and the right TG tissues were extracted 7 days after CFA or saline injection. The TGs were dissociated using the papain dissociation system (Worthington, Lakewood, NJ, United States), according to the manufacturer’s instructions. The right TG tissues were immediately extracted and placed in a pre-cooled papain solution. The tissues were slightly minced, or cut into small pieces, and incubated in the solution at 37°C with constant agitation for 30 to 90 min. The mixture was centrifuged at 300 × *g* for 5 min at room temperature, and the cell pellet was resuspended in DNase dilute albumin-ovomucoid inhibitor solution to stop digestion. The cell density gradient separation solution was prepared by adding 5 ml papain inhibitor to a centrifuge tube and carefully overlaying a layer of the resuspended cell mixture on top of it. Then, the solution was centrifuged at 70 × *g* for 6 min at room temperature, and the cell pellet was resuspended in complete medium to obtain single-cell suspension.

### Single-Cell RNA-Sequencing

The obtained single-cell suspensions were further processed with the BD Rhapsody Single-Cell Analysis System (BD, United States), according to the manufacturer’s protocols, to generate cDNA libraries. Libraries were sequenced utilizing the Illumina NovaSeq platform. Sequence data were submitted to the GEO database (GSE186421). The TG neuronal clusters and their transcriptional profiles were identified and analyzed in both groups.

### Unsupervised Cell Clustering and Uniform Manifold Approximation and Projection (UMAP) Visualization

Unsupervised cell clustering was performed using the Seurat package (version 3.1.2) implemented in R. We filtered out genes expressed in less than two cells, and further excluded cells with expression of <150 genes and >25% mitochondrial genes. Seurat arithmetic was used to calculate the coefficient of variation (CV) for each gene. Principal component analysis (PCA) was performed to reduce the dimensionality of all the data. A k-nearest neighbor graph was constructed based on Euclidean distances in the PCA space. The Louvain modularity optimization algorithm and UMAP visualization were applied to the cell cluster and visualization, respectively.

### Marker Gene Detection and Cell Type Annotation

The Seurat Find Markers function was used to identify the marker genes of each cluster through filtration with log_2_ fold-change (FC) >3, pct >0.5 and *P* < 0.001. The various single-cell datasets were annotated based on previous studies and annotation tool SingleR.

### Bioinformatics Analysis

Differential gene expression analysis was performed to identify DEGs between the control and experimental groups (*P* < 0.05, log_2_|FC∣ ≥ 0.2). Gene Ontology (GO) term enrichment analysis was performed on the identified DEGs for gene annotation and functional enrichment analysis, using clusterProfiler (version 4.2.2)^1^. The GO terms with *P* < 0.05 were identified as significantly enriched for the obtained DEGs. The protein–protein interaction (PPI) network of the proteins encoded by the identified DEGs was constructed using STRING online software^2^ (Szklarczyk et al., 2021), and *P* < 0.05 was considered statistically significant.

### Statistical Analysis

Data are presented as the mean ± standard deviation (SD). The results of mouse mechanical pain thresholds were evaluated by repeated measures ANOVA, while the results of c-Fos expression level were evaluated by unpaired *t*-test. Significance was set at *P* < 0.05, and statistical tests were performed using GraphPad Prism 8. The statistical analyses of marker gene detection and differential gene expression were performed by Wilcoxon Rank Sum test. And statistical significance for marker gene detection analysis was set at *P* < 0.001, while significance for differential gene expression analysis was set at *P* < 0.05. The result of GO term enrichment analysis was calculated by hypergeometric distribution, and *P* < 0.05 was defined to be statistically significant.

## Results

### Orofacial Inflammatory Pain Induced by Complete Freund’s Adjuvant Injection

**FIGURE 1:**
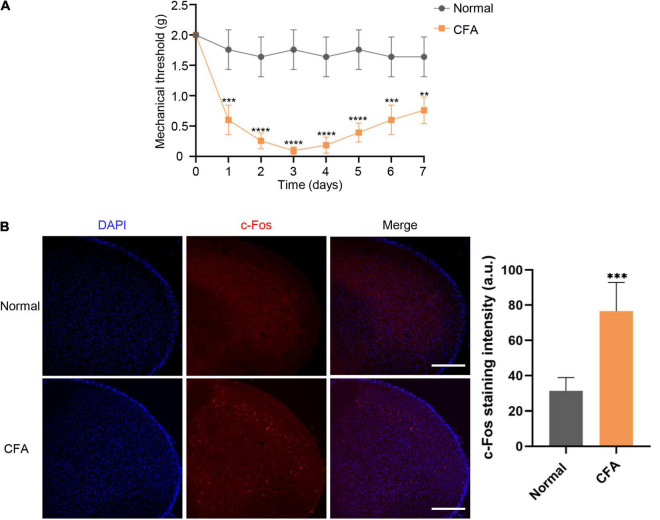
Mechanical thresholds and c-Fos expression in STN following CFA injection. **(A)** Changes in mechanical thresholds of mice before and 7 days after CFA injection. Mean ± SD, *n* = 6, **P < 0.01, ***P < 0.001, ****P < 0.0001. Data were analyzed by repeated measures ANOVA. **(B)** The c-Fos expression alteration in STN under normal and orofacial inflammatory pain conditions. Compared with normal group, the expression level of c-Fos protein in experimental group was significantly increased at 7 days after CFA injection. The blue fluorescence represents DAPI while red fluorescence represents c-Fos. Data were analyzed by unpaired *t*-test. ***P < 0.001, Scale bar = 300 μm. STN, spinal trigeminal nucleus; CFA, complete Freund’s adjuvant.

We performed the Von Frey test to examine the mechanical thresholds in response to mechanical stimuli in mice from both normal and experimental groups. The orofacial mechanical thresholds of CFA-induced mice were significantly decreased compared with the saline-administrated group starting from 1 day after CFA injection. Orofacial mechanical allodynia reached its peak on the third days after CFA injection (*P* < 0.0001), as the threshold alteration was lower by 7 days (*P* < 0.01) (Figure 1A). Immunofluorescent staining was performed to analyze c-Fos expression, a validated marker of neuronal activity, in the STN. Compared with normal group, the expression level of c-Fos protein in experimental group was significantly increased at 7 days after CFA injection (*P* < 0.001) (Figure 1B).

### Profiling Cell Types in Mouse Trigeminal Ganglion by Single-Cell RNA-Sequencing

**FIGURE 2:**
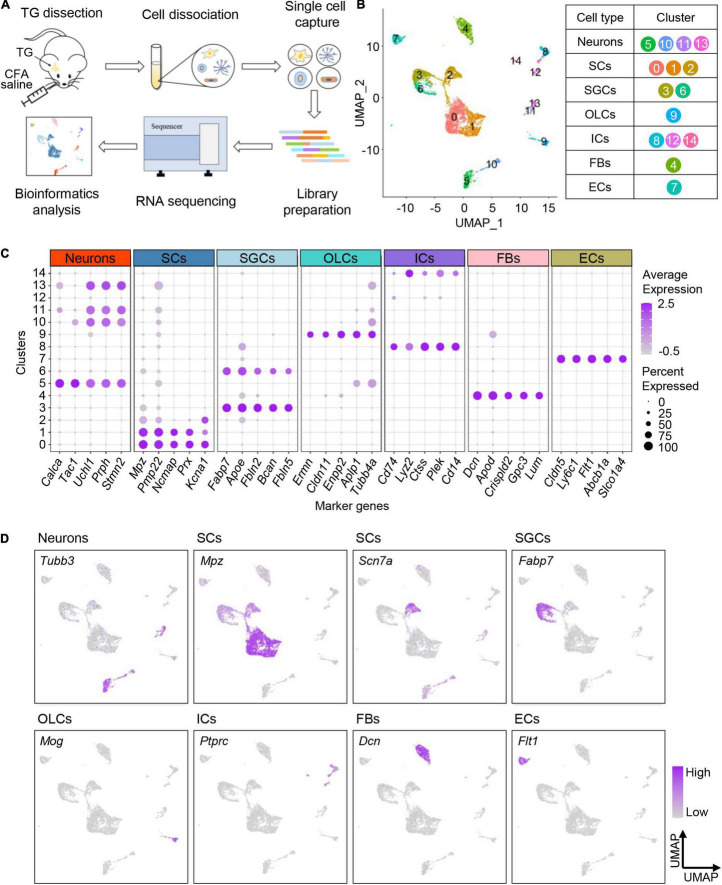
Profiling cell types in mouse TG by scRNA-seq. **(A)** Schematic diagram of experimental design. TGs are dissociated 7 days after CFA/saline injection, and the obtained cell suspensions are further processed with BD Rhapsody scRNA-seq. **(B)** UMAP plot of all cells in TGs obtained from normal and experimental groups. The identified 15 clusters are colored and annotated based on known marker genes from previous studies. **(C)** Dot plot of the top five marker genes for each cell type. Dot color intensity represents the average expression level, and dot size represents the percent of cells expressing the gene. **(D)** UMAP plots of expression pattern of marker genes that best identify each cell type. Marker genes: *Tubb3* (neurons), *Mpz* (myelinating Schwann cells), *Scn7a* (non-myelinating Schwann cells), *Fabp7* (satellite glial cells), *Mog* (oligodendrocyte-like cells), *Ptprc* (immune cells), *Dcn* (fibroblasts), and *Flt1* (endothelial cells). The color intensity represents the expression level. scRNA-seq, single-cell RNA-sequencing; TG, trigeminal ganglion; CFA, complete Freund’s adjuvant; UMAP, uniform manifold approximation and projection; SCs, Schwann cells; SGCs, satellite glial cells; OLCs, oligodendrocyte-like cells; ICs, immune cells; FBs, fibroblasts; ECs, endothelial cells.

To determine the transcriptional profiles of TG neurons in adult mice under orofacial inflammatory pain conditions, we performed BD Rhapsody scRNA-seq on disassociated TG cells 7 days after saline or CFA injection (Figure 2A). We detected 10,187 cells after excluding low-quality cells. On average, each single-cell library detected at least 18,524 reads per cell, revealing at least 1,378 genes per cell. This depth either matches or exceeds that of similar studies (Renthal et al., 2020; Avraham et al., 2021). For homogeneously analysis, we randomly subsampled 3,000 cells per library, 6,000 cells in total (716 neurons included) and further analyzed their transcriptomes (Supplementary Table 1).

To profile cell types residing in TG tissues, TG cells (both normal and experimental groups) were unbiasedly clustered into 15 clusters according to their gene expression patterns, which were visualized with the uniform manifold approximation and projection (UMAP) method (Figure 2B). The identified 15 cell clusters were further annotated using known marker genes from previous studies (Renthal et al., 2020) and annotation tool SingleR (Figure 2B). The cells identified included primary sensory neurons, Schwann cells (SCs), satellite glial cells (SGCs), oligodendrocyte-like cells (OLCs), immune cells (ICs), fibroblasts (FBs), and endothelial cells (ECs) (Figure 2B, Supplementary Figure 1A, and Supplementary Table 2). The expression patterns of top five marker genes and feature genes for each cell type were shown in Figures 2C,D, respectively.

Consistent with previous evidence, neurons specifically expressed *Tubb3*, which encodes beta tubulin and is involved in nervous system formation and maintenance (Latremoliere et al., 2018; Figure 2D). We observed three clusters of glial cells in TG, including Schwann cells, satellite glial cells and a few oligodendrocyte-like cells. Consistent with previous findings (Renthal et al., 2020), myelinating and non-myelinating Schwann cells were characterized by the expression of *Mpz* and *Scn7a*, respectively (Figure 2D). Satellite glial cells that surround the TG neurons were found to specifically express *Fabp7* (Li et al., 2016; Figure 2D). Additionally, oligodendrocyte-like cells highly expressed *Mog*, similar to central oligodendrocytes, but the detailed cellular characteristics require further exploration. Besides, we also identified immune cells expressing *Ptprc*, fibroblasts expressing *Dcn*, and endothelial cells expressing *Flt1*.

### Classifying the Trigeminal Ganglion Neuronal Subtypes by Single-Cell RNA-Sequencing

Traditionally, TG neurons are classified according to their anatomical, genetic, physiological, and biochemical characteristics, however, cellular heterogeneity of TG neurons are ignored due to the technical difficulties. Recent advances in scRNA-seq provide us opportunities to explore the intrinsic heterogeneity of sensory neurons, herein, to explore the intrinsic heterogeneity of sensory neurons, we performed further cluster analysis of TG neurons.

**FIGURE 3:**
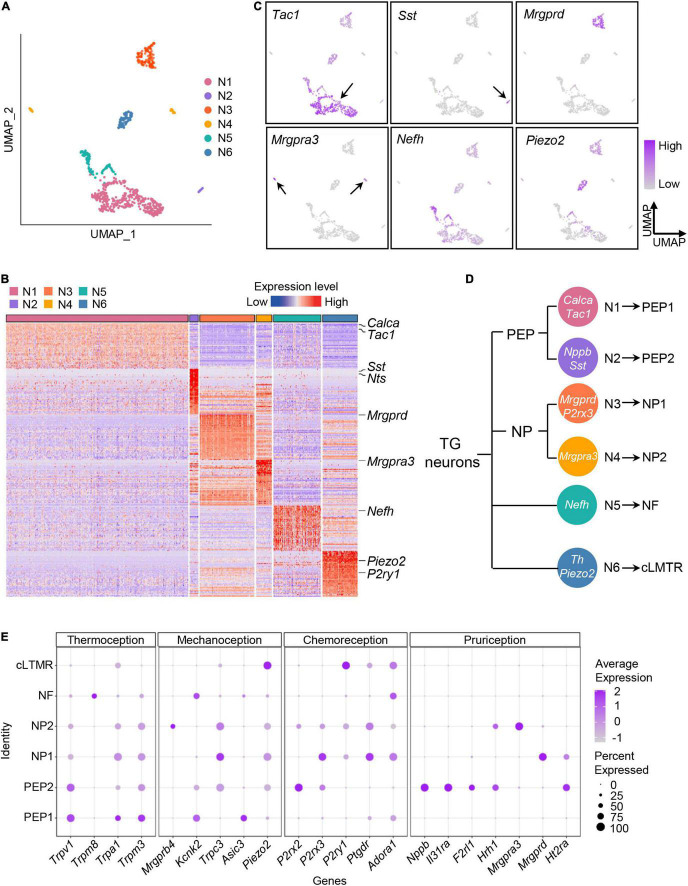
Classifying the TG neuronal subtypes by scRNA-seq. **(A)** UMAP plot of six neuronal subtypes in TGs obtained from normal and experimental groups. **(B)** Heat map of expression of the top 50 significantly enriched genes for each TG neuronal subtype. See Supplementary Table 3 for summary of TG neuronal subgroup expression profile for genes presented in this panel. The differentially expressed (*P* < 0.05) marker genes in each TG neuronal population were ranked by the fold change (maximal likelihood estimate), and the expression levels of the top fifty genes in each population were displayed in a heatmap plot. Red colors indicate higher expression level in the heatmap, while blue colors indicate lower expression level. **(C)** UMAP plots of expression pattern of marker genes that best identify each TG neuronal subtype. Marker genes: *Tac1* (N1), *Sst* (N2), *Mrgprd* (N3), *Mrgpra3* (N4), *Nefh* (N5), *Piezo2* (N6). *Tac1* encodes substance P; *Sst*, somatostatin; *Mrgprd*, MRGPRD; *Mrgpra3*, MRGPRA3; *Nefh*, neurofilament heavy chain; *Piezo2*, Piezo type mechanosensitive ion channel component 2. The color intensity represents the expression level. Arrows were used to indicate the location of gene expression. **(D)** Schematic diagram of classification of the TG neurons. The marker genes of TG neuronal subtypes are indicated in white. **(E)** Dot plot of expression pattern of functional molecules in TG neuronal subtypes. Representative functional molecules have been proven to be involved in thermoception, mechanoception, chemoreception and pruriception (Li et al., 2016). Dot color intensity represents the average expression level, and dot size represents the percent of cells expressing the gene. scRNA-seq, single-cell RNA-sequencing; UMAP, uniform manifold approximation and projection; TG, trigeminal ganglion; PEP, peptidergic nociceptors; NP, non-peptidergic nociceptors; NF, *Nefh*-positive neurons; cLTMR, C-fiber low-threshold mechanoreceptors.

As shown in Figure 3A, TG neurons were unbiasedly clustered into six categories (Neuron 1–6). Based on expression patterns of differentially expressed (*P* < 0.05) marker genes and feature genes in each population (Figures 3B,C), Neuron 1 and Neuron 2 were identified as peptidergic nociceptors (PEP), Neuron 3 and 4 as non-peptidergic nociceptors (NP), Neuron 5 as myelinated neurons (*Nefh*-positive neurons, NF), and Neuron 6 as C-fiber low-threshold mechanoreceptors (cLTMR) (Figure 3D). In detail, we identified that Neuron 1 showed expression of *Tac1* (encoding substance P) and *Calca* (encoding calcitonin gene-related peptide), which was later labeled as PEP1 (Figure 3C and Supplementary Figure 1B), meanwhile, Neuron 2 were specifically expressed *Sst* (encoding somatostatin) and *Nppb* (encoding natriuretic peptide B), which was later labeled as PEP2 (Figure 3C and Supplementary Figure 1B). For NP neurons, we identified Neuron 3 population was defined by expression of the marker *Mrgprd* and *P2rx3* and was labeled as NP1, meanwhile, Neuron 4 population was defined by expression of itch-related gene *Mrgpra3* and was named as NP2 (Figure 3C). *Nefh* (encoding neurofilament heavy chain) was assigned as marker gene for NF neurons, meanwhile, *Piezo2* and *Th* as marker genes for cLTMR (Figure 2C and Supplementary Figure 1B). Marker genes for each TG neuronal subtypes were summarized in schematic diagram (Figure 3D).

To explore the potential sensory modalities between distinct TG neuronal subtypes, we performed analysis of functional molecules in neuron populations based on previous reports (Li et al., 2016). We found that PEP1 highly expressed genes related to thermoception and mechanoception. For example, *Trpv1*, detector of high temperatures and pungent chemicals, was highly expressed in PEP1 (Figure 3E). *Trpa1* was mainly expressed in PEP1, which has been reported to be activated by cold and mechanical stimuli (Talavera et al., 2020; Figure 3E). Additionally, the heat-activated ion channel *Trpm3* was also highly present in PEP1 (Figure 3E). The genes encoding mechanoreceptive molecules, such as *Kcnk2* and *Asic3*, were also expressed in PEP1 (Figure 3E). For PEP2, it expressed high levels of *Trpv1* and purinergic receptor gene *P2rx2*. Notably, PEP2 neurons were more likely to be implicated in itch perception according to the high levels of pruritogen receptors in PEP2. For example, PEP2 neurons contained mRNAs encoding the interleukin 31 receptor A (*Il31ra*) and histamine H1 receptor (*Hrh1*), and therefore could be specific for interleukin 31- and histamine-evoked itch (Datsi et al., 2021; Figure 3E). For NP1, it expressed various functional molecules, such as mechanoreceptive molecule gene *Trpc3*, purinergic receptor genes *P2rx3* and *P2ry1*, and pruritogen receptor *Mrgprd*. For NP2, it expressed high level of *Mrgpra3* (encoding mas-related G-coupled protein receptor) (Figure 3E), which is involved in perception of itch and pain. For NF, it specifically expressed *Trpm8*, which is associated with thermosensation over a wide range of cold temperatures (Bautista et al., 2007; Figure 3E). For cLTMR, it highly expressed mechanically activated ion channel *Piezo2*, which has a role in mechanosensation and nociception (Figure 3E).

### Transcriptional Responses of Trigeminal Ganglion Neuronal Subtypes to Orofacial Inflammation Revealed by Single-Cell RNA-Sequencing

**FIGURE 4:**
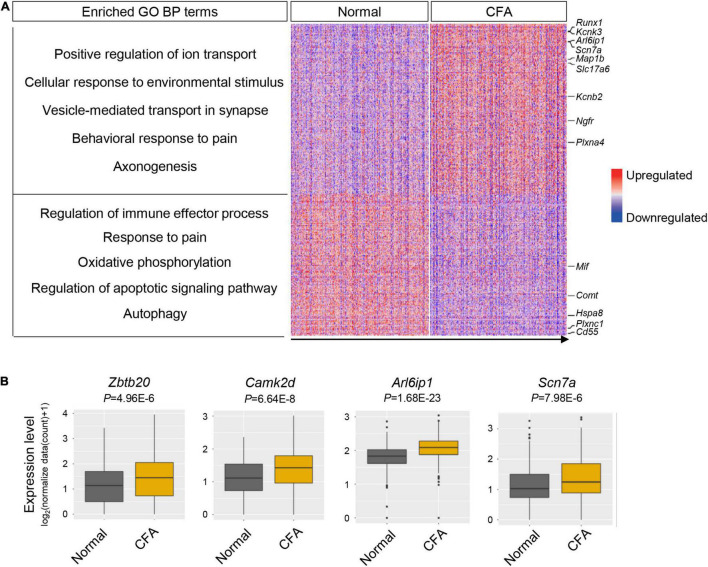
Transcriptional alterations of overall TG neurons in response to orofacial inflammation. **(A)** Heatmap and enriched GO biological process terms of identified DEGs with overall TG neurons upon orofacial inflammatory pain. A total of 488 DEGs were identified, including 266 upregulated and 222 downregulated genes (*P* < 0.05, log_2_| FC| ≥ 0.2, Supplementary Table 4). The identified DEGs were associated with the inflammatory response (such as *Cd55*, *Plxnc1*, and *Mif*), neuronal excitability and plasticity (such as *Arl6ip1*, *Scn7a*, *Kcnb2*, and *Kcnk3*), neuroregeneration and neuroprotection (such as *Ngfr*, *Map1b*, and *Plxna4*), and mitochondrial function and autophagy (such as *mt-Nd6* and *Hspa8*). Red colors indicate upregulated expression level in the heatmap, while blue colors indicate downregulated expression level. **(B)** Boxplots of the expression of *Zbtb20*, *Camk2d, Arl6ip1*, and *Scn7a* in overall TG neurons under normal and orofacial inflammatory pain conditions, which are likely to participate in pain modulation. TG, trigeminal ganglion; DEGs, differentially expressed genes; FC, fold change; CFA, complete Freund’s adjuvant.

To explore the transcriptional alterations of TG neurons in response to orofacial inflammation, we performed differential gene expression analysis between normal group and CFA group. A total of 488 DEGs were identified within TG neuronal populations, including 266 upregulated and 222 downregulated genes (*P* < 0.05, log_2_∣FC∣ ≥ 0.2, Supplementary Table 4), which were shown in heatmap (Figure 4A). Differential gene expression analysis revealed several DEGs in TG neurons that were previously reported to regulate inflammatory response (including *Cd55*, *Plxnc1*, and *Mif*), neuronal excitability and plasticity (such as *Arl6ip1*, *Scn7a, Kcnb2*, and *Kcnk3*), neuroregeneration and neuroprotection (such as *Ngfr*, *Map1b*, and *Plxna4*) as well as genes that regulate mitochondrial function, autophagy, and apoptosis (such as *mt-Nd6* and *Hspa8*). However, a large amount of DEGs detected in our date set (Figure 4B), which may play essential roles in neuron activities and regulate inflammatory pain, require further verification. Correspondingly, GO enrichment analysis of identified DEGs further revealed several enriched biological process (BP) terms (Figure 4A and Supplementary Table 5), including “positive regulation of ion transport,” “regulation of immune effector process,” “response to pain,” and “autophagy,” implying the dynamic changes in neuronal activities and diverse roles of TG neurons in response to orofacial inflammation.

It has been proposed that distinct TG neuronal subtypes play specific roles in orofacial pain and show unique transcriptional patterns (Sun and Chen, 2016). In order to investigate heterogeneous responses and assess the functions of each subtype under orofacial inflammatory pain condition, we investigated the transcriptomes of each TG neuronal subtype (PEP1, PEP2, NP1 NP2, cLTMR, and NF) in our current dataset. Differential gene expression and GO enrichment analysis were carried out on the TG neuronal subpopulations. Additionally, to further investigate the relationships between the DEGs under orofacial inflammatory pain, the PPI networks were constructed using STRING, and hub genes were identified using Cytoscape.

#### PEP1 and PEP2

**FIGURE 5:**
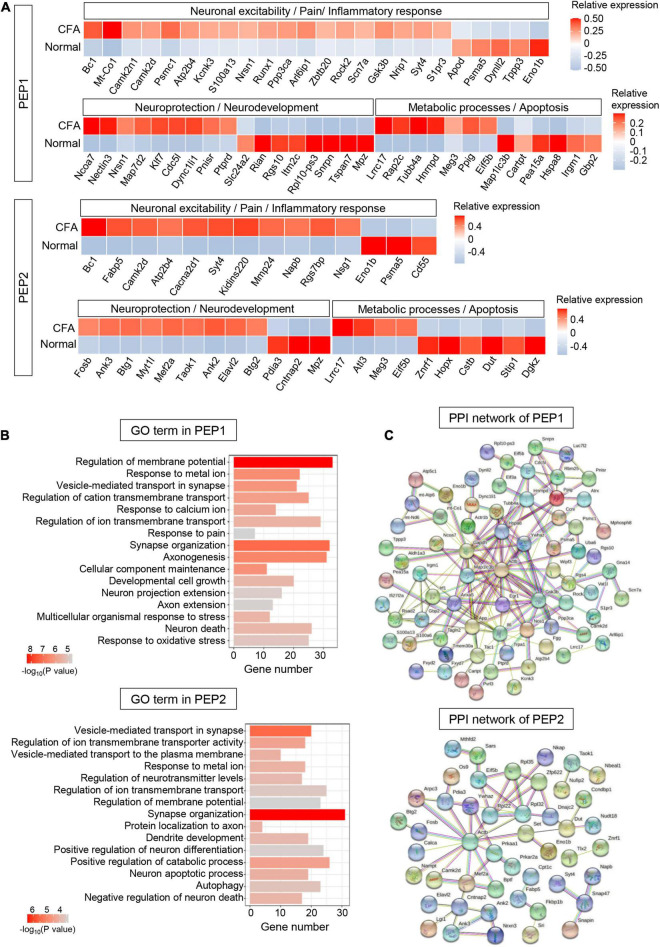
Transcriptional responses of PEP neurons in mouse TG to orofacial inflammation revealed by scRNA-seq. **(A)** Heatmap of identified DEGs within PEP1 and PEP2 neurons under normal and orofacial inflammatory pain conditions. Red colors indicate upregulated expression level in the heatmap, while blue colors indicate downregulated expression level. **(B)** The enriched GO biological process terms of identified DEGs in PEP1 and PEP2 neurons following orofacial inflammation. The number of genes is plotted as the abscissa and enriched GO biological process term is plotted as the ordinate. The colors correspond to *p*-value range. **(C)** PPI network analysis of identified DEGs in PEP1 and PEP2 neurons following orofacial inflammation. The top hub genes in PEP1 are *Actb* (degree = 22), *Il6* (degree = 17), *Gsk3b* (degree = 13), *Hspa8* (degree = 12), *Map1lc3b* (degree = 10), and *Nos1* (degree = 9), while top hub genes in PEP2 are *Actb* (degree = 13), *Gsk3b* (degree = 14), *Rpl32* (degree = 7), *Eif5b* (degree = 4), and *Ywhaz* (degree = 4), etc. The line color indicates the type of interaction evidence, and line thickness indicates the strength of data support. Blue represents known interactions from curated databases, while purple represents experimentally determined interactions. Green, red, and royal blue represent gene neighborhood, gene fusions and gene co-occurrence, respectively. TG, trigeminal ganglion; scRNA-seq, single-cell RNA-sequencing; DEGs, differentially expressed genes; CFA, complete Freund’s adjuvant; PEP, peptidergic nociceptors; PPI, protein–protein interaction.

Five hundred sixty-six DEGs (including 313 upregulated and 253 downregulated genes) were detected in PEP1 (*P* < 0.05, log_2_∣FC∣ ≥ 0.2, Supplementary Table 6). In top 100 DEGs, we identified several genes regulating neuronal excitability and plasticity in PEPs, such as *Bc1* and *Camk2d* (Figure 5A). Notably, we identified various pain-related genes essential for neuronal signal transduction in PEP1, including ion channel genes *Kcnk3* and *Scn7a*, and glutamate signaling-related genes *Arl6ip1* and *Gsk3b* (Figure 5A). Correspondingly, GO enrichment analysis of DEGs revealed that PEP1 may be involved in regulating neuronal excitability, pain sensation, and biological processes such as “regulation of membrane potential,” “response to metal ion,” “vesicle-mediated transport in synapse,” and “response to pain” (Figure 5B and Supplementary Table 6). Notably, more than 20 genes were enriched in several neuronal excitability and pain-related GO terms, which indicates the possible role of PEP1 in neuronal excitation and nociception. PPI network of DEGs was established to indicate the molecule interactions in PEP1, which revealed 91 nodes (hub genes) and 154 edges (interactions) in PEP1 (PPI enrichment *P* < 3.89e-09) (Figure 5C and Supplementary Table 6). The top hub genes with high connectivity degrees in PEP1 neurons were *Actb* (degree = 22), *Il6* (degree = 17), *Gsk3b* (degree = 13), *Hspa8* (degree = 12), *Map1lc3b* (degree = 10), and *Nos1* (degree = 9), some of which have been indicated to be implicated in pain regulation.

Five hundred eighty-eight DEGs (including 267 upregulated and 321 downregulated genes) were detected in PEP2 (*P* < 0.05, log_2_∣FC∣ ≥ 0.2, Supplementary Table 7). In top 100 DEGs, we identified DEGs related to inflammatory response, like *Fabp5* and *Cd55*. The expression of Ca^2+^ signaling-related genes *Cacna2d1* and *Kidins220* were increased in PEP2 (Figure 5A). Furthermore, the expression level of *Rgs7bp* was enhanced in PEP2, which has been demonstrated to play a role in the regulation of itch sensation. Several DEGs for neuroprotection (such as *Ank3* and *Mef2a*), and neuronal degeneration (such as *Atl3, Meg3*, and *Znrf1*) were also detected in PEP2. GO enrichment analysis revealed that DEGs in PEP2 were enriched in “vesicle-mediated transport in synapse,” “response to metal ion,” and “regulation of neurotransmitter levels,” notably, more than 30 genes were enriched in synapse organization, which indicates the possible role of PEP2 in neuronal excitation and neuroprotection (Figure 5B and Supplementary Table 7). The established PPI network contained 93 nodes and 59 edges in PEP2 (PPI enrichment *P* < 0.01) (Figure 5C and Supplementary Table 7). Additionally, *Actb* (degree = 13), *Rpl32* (degree = 7), *Eif5b* (degree = 4), and *Ywhaz* (degree = 4), etc., were selected as hub genes in the PEP2 network. So, the DEGs in PEP2 may have effects on nociception and itch sensation, but further in-depth studies are still necessary.

#### NP1 and NP2

**FIGURE 6:**
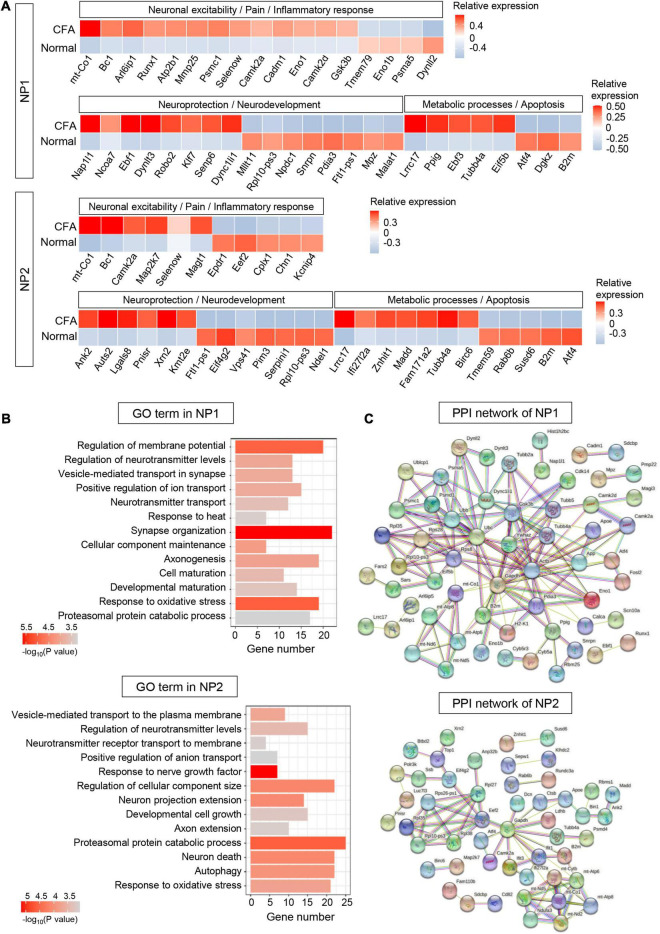
Transcriptional responses of NP neurons in mouse TG to orofacial inflammation revealed by scRNA-seq. **(A)** Heatmap of identified DEGs within NP1 and NP2 neurons under normal and orofacial inflammatory pain conditions. Red colors indicate upregulated expression level in the heatmap, while blue colors indicate downregulated expression level. **(B)** The enriched GO biological process terms of identified DEGs in NP1 and NP2 neurons following orofacial inflammation. The number of genes is plotted as the abscissa and enriched GO biological process term is plotted as the ordinate. The colors correspond to *p*-value range. **(C)** PPI network analysis of identified DEGs in NP1 and NP2 neurons following orofacial inflammation. The top hub genes in NP1 are *Actb* (degree = 23), *Ubc* (degree = 15), *Ywhaz* (degree = 12), *Gsk3b* (degree = 11), and *mt-Co1* (degree = 7), while top hub genes in NP2 are *Gapdh* (degree = 14), *Eef2* (degree = 7), *mt-Atp6* (degree = 7), *mt-Co1* (degree = 7), and *Rpl10-ps3* (degree = 5), etc. The line color indicates the type of interaction evidence, and line thickness indicates the strength of data support. Blue represents known interactions from curated databases, while purple represents experimentally determined interactions. Green, red, and royal blue represent gene neighborhood, gene fusions and gene co-occurrence, respectively. TG, trigeminal ganglion; scRNA-seq, single-cell RNA-sequencing; DEGs, differentially expressed genes; CFA, complete Freund’s adjuvant; NP, non-peptidergic nociceptors; PPI, protein–protein interaction.

Three hundred sixty-nine DEGs (including 201 upregulated and 168 downregulated genes) were detected in NP1 (*P* < 0.05, log_2_∣FC∣ ≥ 0.2, Supplementary Table 8). In top 100 DEGs, we identified many genes associated with inflammatory response (such as *Cadm1* and *Mmp25*), neuronal excitability (such as glutamate signaling-related genes *Arl6ip1* and *Gsk3b*), and neuroprotection and neurodevelopment (such as *Nap1l1* and *Ebf1*) in NP1 (Figure 6A). Based on the GO enrichment analysis, identified DEGs in NP1 were significantly enriched in “regulation of membrane potential,” “positive regulation of ion transport,” “response to heat,” “synapse organization,” and “axonogenesis,” which indicates the possible role of NP1 in regulating neuronal excitability and neuroprotection (Figure 6B and Supplementary Table 8). The established PPI network contained 90 nodes and 144 edges in NP1 (PPI enrichment *P* < 3.42e-06) (Figure 6C and Supplementary Table 8). The top hub genes with high connectivity degrees in NP1 neurons were *Actb* (degree = 23), *Ubc* (degree = 15), *Ywhaz* (degree = 12), *Gsk3b* (degree = 11), and *mt-Co1* (degree = 7), some of which are associated with glutamate transport.

Five hundred thirty-nine DEGs (including 175 upregulated and 364 downregulated genes) were detected in NP2 (*P* < 0.05, log_2_∣FC∣ ≥ 0.2, Supplementary Table 9). In NP2 neurons, we identified many DEGs related to neuroprotection (such as *Ank2* and *Lgals8*) and cellular metabolism (such as *Lrrc17* and *Atf4*) (Figure 6A). Correspondingly, GO enrichment analysis demonstrated that DEGs in NP2 were significantly enriched in “response to nerve growth factor,” “proteasomal protein catabolic process,” and “neuron death” (Figure 6B and Supplementary Table 9). Notably, more than 20 genes were enriched in “proteasomal protein catabolic process” and “response to oxidative stress.” Therefore, NP2 neurons may have valuable functions in neuroprotection and metabolic process. Then, the established PPI network contained 92 nodes and 75 edges in NP2 (PPI enrichment *P* < 4.62e-05) (Figure 6C and Supplementary Table 9). Additionally, *Gapdh* (degree = 14), *Eef2* (degree = 7), *mt-Atp6* (degree = 7), *mt-Co1* (degree = 7), and *Rpl10-ps3* (degree = 5), etc., were selected as hub genes in the NP2 network.

#### C-Fiber Low-Threshold Mechanoreceptors and *Nefh*-Positive Neurons

**FIGURE 7:**
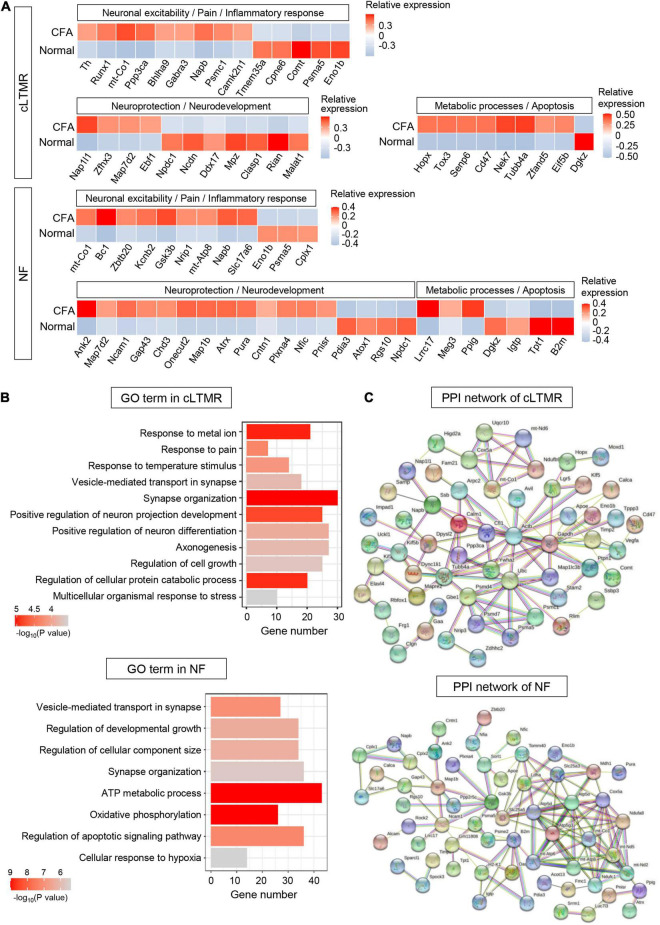
Transcriptional responses of cLTMR and NF neurons in mouse TG to orofacial inflammation revealed by scRNA-seq. **(A)** Heatmap of identified DEGs within cLTMR and NF neurons under normal and orofacial inflammatory pain conditions. Red colors indicate upregulated expression level in the heatmap, while blue colors indicate downregulated expression level. **(B)** The enriched GO biological process terms of identified DEGs in cLTMR and NF neurons following orofacial inflammation. The number of genes is plotted as the abscissa and enriched GO biological process term is plotted as the ordinate. The colors correspond to *p*-value range. **(C)** PPI network analysis of identified DEGs in cLTMR and NF neurons following orofacial inflammation. The top hub genes in cLTMR are *Actb* (degree = 18), *Ubc* (degree = 11), *Ywhaz* (degree = 7), *Calm1* (degree = 6), *mt-Co1* (degree = 6), and *Cfl1* (degree = 6), while top hub genes in NF are *Atp5d* (degree = 12), *Cox5a* (degree = 12), *Atp5g3* (degree = 11), *mt-Co1* (degree = 10), and *B2m* (degree = 8), etc. The line color indicates the type of interaction evidence, and line thickness indicates the strength of data support. Blue represents known interactions from curated databases, while purple represents experimentally determined interactions. Green, red, and royal blue represent gene neighborhood, gene fusions and gene co-occurrence, respectively. TG, trigeminal ganglion; scRNA-seq, single-cell RNA-sequencing; DEGs, differentially expressed genes; CFA, complete Freund’s adjuvant; NF, *Nefh*-positive neurons; cLTMR, C-fiber low-threshold mechanoreceptors; PPI, protein–protein interaction.

Six hundred forty-nine DEGs (including 332 upregulated and 317 downregulated genes) were detected in cLTMR (*P* < 0.05, log_2_∣FC∣ ≥ 0.2, Supplementary Table 10). In top 100 DEGs, we found some genes related to neuronal excitability and pain regulation in cLTMR neurons, such as *Runx1*, *Napb*, and *Comt*. Notably, the transcription factor gene *Bhlha9* important for temperature sensation and pain modulation was significantly upregulated in cLTMR (Figure 7A). Similarly, identified DEGs in cLTMR were significantly enriched in “response to metal ion,” “response to pain,” “response to temperature stimulus,” and “positive regulation of neuron projection development” (Figure 7B and Supplementary Table 10), which indicates the important role of cLTMR in nociception. Then, the established PPI network contained 90 nodes and 103 edges in cLTMR (PPI enrichment *P* < 2e-05) (Figure 7C and Supplementary Table 10). The top hub genes with high connectivity degrees in cLTMR neurons were *Actb* (degree = 18), *Ubc* (degree = 11), *Ywhaz* (degree = 7), *Calm1* (degree = 6), *mt-Co1* (degree = 6), and *Cfl1* (degree = 6), some of which are involved in synaptic transmission.

Eight hundred five DEGs (including 339 upregulated and 466 downregulated genes) were detected in NF (*P* < 0.05, log_2_∣FC∣ ≥ 0.2, Supplementary Table 11). In NF neurons, we identified many DEGs involved in neuroprotection (such as *Ank2* and *Map7d2*) and metabolic process (such as *Lrrc17* and *Dgkz*) (Figure 7A). Similarly, DEGs in NF were enriched in “ATP metabolic process,” “oxidative phosphorylation,” and “regulation of developmental growth” (Figure 7B and Supplementary Table 11). Notably, more than 40 genes were enriched in “ATP metabolic process,” which indicates the important role of NF in metabolic process. The established PPI network contained 94 nodes and 125 edges in NF (PPI enrichment *P* < 1.0e-16) (Figure 7C and Supplementary Table 11). Additionally, *Atp5d* (degree = 12), *Cox5a* (degree = 12), *Atp5g3* (degree = 11), *mt-Co1* (degree = 10), and *B2m* (degree = 8), etc., were selected as hub genes in the NF network, many of which are associated with ATP synthesis.

## Discussion

Different subtypes of primary sensory neurons play distinct roles in the basal sensation and pathogenesis of pain. It remains largely unclear about the diverse alterations and regulatory mechanisms exhibited by different types of TG neurons in response to orofacial inflammatory pain. Mapping neuronal transcriptional alterations and investigating the molecular characteristics of distinct neurons are critical for understanding their roles in pain initiation and maintenance. A previous RNA-sequencing study investigated the molecular composition of small DRG nociceptive neurons using magnetic purification, but it is difficult to determine the source of genes expressed at lower levels in neurons (Thakur et al., 2014). Recent advances in scRNA-seq techniques allow the detection of gene profiles of single cells under pathological conditions, providing a remarkable opportunity to understand disease mechanisms (Ofengeim et al., 2017; Armand et al., 2021). Chronic pain is pain that lasts more than 3 months (Treede et al., 2015), in this study, we selected 7 days after CFA injection in mice as one of typical time points to investigate preliminarily the mechanisms of chronic orofacial pain in humans (Dutta and Sengupta, 2016). After scRNA-seq analysis, we unbiasedly classified six clusters of TG sensory neurons based on their transcriptional characteristics, which were later assigned as PEP1, PEP2, NP1, NP2, cLTMR, and NF. Most importantly, we investigated the transcriptomes of the TG neuronal subtypes under orofacial inflammatory conditions and interpretated their possible roles using bioinformatic methods, which not only comprehensively profiles the cellular transcriptional responses of TG neurons to orofacial inflammation but also provides several potential new targets for pain control.

### Novel Classification of Trigeminal Ganglion Sensory Neurons With Single-Cell RNA-Sequencing

Neurons exhibit diverse molecular, morphological, connectional, and functional properties (Zeng and Sanes, 2017). Previous studies classified primary sensory neurons according to anatomical (Boycott and Wassle, 1991), molecular (Kodama et al., 2012) and electrophysiological criteria (Markram et al., 2004). However, the deficiency of these approaches is that the heterogeneity of cells cannot be reflected. In the present study, we classified TG sensory neurons into six subpopulations (including two types of PEP neurons, two types of NP neurons, NF, and cLTMR) using scRNA-seq based on transcriptome patterns, which revealed the cellular heterogeneity in TG. And there are also slightly different from the historical classifications. For instance, there was co-expression of *Th* and *Piezo2* in cLTMR neurons in this study. And the cross-cluster expressions of *Tac1* and *Calca* are not limited to PEP neurons, which is consistent with previous studies (Li et al., 2016).

Notably, the differential expression of marker genes in distinct neurons implies the functionality of these neuron clusters. To explore the functions of each subtype, analysis of functional molecules in neuron populations was conducted. We found PEP1 neurons were likely to play vital roles in temperature sensation and mechanoception perception based on the high expressions of TRP channels (such as *Trpv1*, *Trpa1*, and *Trpm3*) and mechanoreceptive molecules (such as *Kcnk2* and *Asic3*), which are involved in regulating nociception (Dhaka et al., 2006; Julius, 2013). Research has indicated that TRPA1 is associated with the transduction of mechanical, cold, and chemical stimuli in nociceptors, while TRPV1 is a thermosensitive ion channel that can be activated by various noxious stimuli, resulting in pain, itching, and burning sensation in inflammatory tissue (Kwan et al., 2006; Moore et al., 2018). Extensive studies have demonstrated that TRPA1 and TRPV1 channels are highly expressed in small and medium nociceptor neurons of the DRG and TG (Moore et al., 2018), in the present study, *Trpa1* and *Trpv1* were expressed at high levels in PEP1. Consistent with previous findings (Yajima et al., 2019), *Trpm3* showed high co-existence with *Trpv1* in PEP1, which probably plays a role in thermosensitive nociception. The presence of mechanosensitive channel *Kcnk2* and acid sensing ion channel *Asic3* in PEP1 may be associated with peripheral nociception (Cohen et al., 2009; Deval and Lingueglia, 2015; Lin et al., 2015). Meanwhile, we identified PEP2 neurons can be implicated in participating in pruriceptive responses according to the high levels of itch perception molecules (such as *Nppb*, *Il31ra, and Hrh1*) in PEP2 (Sharif et al., 2020; Datsi et al., 2021). For NP1, it expressed various functional molecules, such as mechanoreceptive molecule like *Trpc3* and purinergic receptor such as *P2rx3* and *P2ry1*. Accumulating evidence has also revealed the key role of mechanosensitive channel *Trpc3* in nociception mediation (Quick et al., 2012) and purinergic signaling in chemoreception and orofacial pain (Lim and Mitchell, 2012; Sobrinho et al., 2014; Moreira et al., 2015; Luo et al., 2022). The high level of *Mrgpra3* in NP2 implies its effect on perception of itch and pain (Liu et al., 2009; Xing et al., 2020; Grundy et al., 2021). The specific expression of *Trpm8* in NF can contribute to sensing unpleasant cold stimuli or mediating the effects of cold analgesia (Dhaka et al., 2007; Dai, 2016; De Caro et al., 2019). As the primary mechanotransduction ion channel, *Piezo2* was expressed highly in cLTMR, promoting its critical role in touch sensation and tactile pain (Ranade et al., 2014; Szczot et al., 2018). Thus, the present study provides the classification and predicted functionality of TG neurons by transcriptome analysis.

### Gene Regulation in Orofacial Inflammatory Pain

Orofacial hypersensitivity is caused by alterations in peripheral inflammation-induced gene expression in TG neurons. In the present study, we further confirmed the different transcriptional responses of TG neuronal subtypes following orofacial inflammation, providing a rational basis for the development of therapeutic strategies.

Neuronal excitability changes in the TG are considered to be the underlying mechanisms causing orofacial peripheral hypersensitivity associated with orofacial inflammation (Basbaum et al., 2009; Shinoda et al., 2019). Glutamate is the major excitatory neurotransmitter in peripheral neurons and is a strategic target in nociceptive processing triggered by inflammation and peripheral nerve injury (Malet and Brumovsky, 2015). Numerous studies have demonstrated that enhanced glutamatergic transmission contributes to orofacial pain hypersensitivity (Cseh et al., 2020; Li et al., 2020). Here, we identified significantly increased *Slc17a6* expression, which encodes vesicular glutamate transporter type 2. Notably, we also discovered several upregulated glutamate-related genes, such as *Arl6ip1* and *Gsk3b* in PEP1 and NP1 and *Napb* in PEP2 and cLTMR. *Arl6ip1* and *Gsk3b* can be involved in the formation of hyperalgesia via mediating glutamate transport activity (Akiduki and Ikemoto, 2008; Weng et al., 2014; Deng et al., 2019), while *Napb* is associated with formation of glutamate vesicle, although the precise function and mechanism of these genes in orofacial inflammatory pain deserve further study. Voltage-gated ion channels, particularly sodium and potassium channels, are the principal mediators of neuronal excitability and are closely linked to hyperalgesia (Bennett et al., 2019; Lee et al., 2019). In the present study, we identified upregulated potassium channel *Kcnk3* in PEP1 and calcium channel *Cacna2d1* in PEP2. Noticeably, we also identified upregulated expression of the sodium channel gene *Scn7a* in PEP1, which has been indicated to contribute to cancer pain by increasing excitability of neurons in DRG (Ke et al., 2012). Meanwhile, we found upregulated expression of zinc-finger protein gene, *Zbtb20*, in PEP1 neurons upon orofacial inflammatory pain, which can regulate nociception and pain sensation by modulating TRP channel expression in DRG (Ren et al., 2014). The roles of *Scn7a* and *Zbtb20* obtained from the DRG in pain hypersensitivity could also be operative in the TG, which should be worthy to be further validated, although the pathophysiology of the trigeminal nerve differs from that of spinal nerves to an extent (Lopes et al., 2017). Additionally, consistent with the predicted role of PEP2, the gene *Rgs7bp*, encoding regulator of G protein signaling 7 binding protein, was increased in PEP2 neurons, which has significant effects on the regulation of itch sensation (Pandey et al., 2017). Consistent with recent study, we found the upregulated expression of transcription factor *Bhlha9* in cLTMR, which has vital function in inflammatory formalin pain and temperature sensation (Bohic et al., 2020).

The mechanisms of orofacial inflammatory pain have been studied extensively, predominantly involving sensory neuron-immune interactions (Pinho-Ribeiro et al., 2017). In this study, GO enrichment analysis suggested that the identified DEGs were significantly enriched in ion transport, regulation of immune effector process, response to pain, and neurotransmitter transport, etc. These were also confirmed to be engaged in pain generation under inflammatory conditions (Wood et al., 2004; Pinho-Ribeiro et al., 2017). Additionally, identified DEGs and GO terms were also associated with ATP synthesis, oxidative stress and neuroprotection. Emerging evidence has illustrated that ATP pathway and oxidative stress are critically involved in pain regulation (De Logu et al., 2020; Xu et al., 2021; Luo et al., 2022). Peripheral nerve injury induced by axotomy and TG extraction during our experiments do not rule out the transcriptional alterations of neuroregeneration and neuroprotection genes in neuron. Moreover, the hub genes, such as *Actb*, *Gsk3b*, and *mt-Co1*, were identified, which are closely correlated with pain development (Liu et al., 2017; Noori et al., 2020; Segelcke et al., 2020). And the roles and mechanisms of several potential novel targets in orofacial inflammatory pain, such as *Scn7a* and *Zbtb20* in PEP1, *Rgs7bp* in PEP2, and *Bhlha9* in cLTMR, require further exploration.

Although gene expression in TG neurons changes over time following inflammation, we performed scRNA-seq on TG cells 7 days after orofacial inflammation, due to the limitation of experimental conditions. Tracing the dynamic cellular activities of TG neurons in orofacial pain is of great significance, therefore, it is necessary to further study gene expression alterations in TG neurons over time following inflammation, such as 1, 3, and 14-day after CFA injection. There is growing evidence for sex differences in orofacial pain prevalence, sensitivity and analgesic response (Shaefer et al., 2018), therefore, further explorations that utilize scRNA-seq analysis to unveil gender differences in orofacial pain are needed. Our results illustrated several potential targets of orofacial inflammatory pain, but further verification experiments are necessary. In addition, we did not assume that unchanged transcript level genes have no effect on orofacial pain modulation. Despite these limitations, we believe that transcriptome analysis is a promising approach for predicting the overall neuronal events related to orofacial inflammation and inferring mechanistic cascades leading to peripheral sensitization.

## Conclusion

The present study not only revealed cell heterogeneity of TG neurons, but also identified transcriptomic alterations of TG sensory neurons in response to orofacial inflammation. Our findings lay the foundation for further exploration of the underlying mechanisms of orofacial pain to aid the development of novel approaches in managing such pain conditions.

## Data Availability Statement

The datasets presented in this study can be found in online repositories. The names of the repository/repositories and accession number(s) can be found below: https://www.ncbi.nlm.nih.gov/geo/, GSE186421.

## Ethics Statement

The animal study was reviewed and approved by the Ethics Committee of the Sun Yat-sen University in China.

## Author Contributions

QL and LM designed and drafted the manuscript and figure. SY, SJ, and YC analyzed the data. FH, WF, and HH revised the manuscript. All authors read and approved the final manuscript.

## Conflict of Interest

The authors declare that the research was conducted in the absence of any commercial or financial relationships that could be construed as a potential conflict of interest.

## Publisher’s Note

All claims expressed in this article are solely those of the authors and do not necessarily represent those of their affiliated organizations, or those of the publisher, the editors and the reviewers. Any product that may be evaluated in this article, or claim that may be made by its manufacturer, is not guaranteed or endorsed by the publisher.

## Footnotes

1
https://bioconductor.org/packages/clusterProfiler/

2
https://cn.string-db.org/

## Supplementary Material

Supplementary Figure 1 **(A)** UMAP plot of all cells in mouse TGsobtained from normal and experimental groups. The identified seven types of cells in TG. The proportions of each cell type in different groups are displayed in bar plots and

Supplementary Table 1 **(B)** UMAP plots of expression pattern of marker genes that best identify each TG neuronal subtype. Marker genes: *Calca* (N1/PEP1), *Nppb* (N2/PEP2), *P2rx3* (N3/NP1), *Th* (N6/cLTMR). TG, trigeminal ganglion; CFA, complete Freund’s adjuvant; UMAP, uniform manifold approximation and projection; SCs, Schwann cells; SGCs, satellite glial cells; OLCs, oligodendrocyte-like cells; ICs, immune cells; FBs, fibroblasts; ECs, endothelial cells; PEP, peptidergic nociceptors; NP, non-peptidergic nociceptors; NF, *Nefh*-positive neurons; cLTMR, C-fiber low-threshold mechanoreceptors. Arrow was used to indicate the location of gene expression.
