# Supplementary figures and images for: Transcriptional Alterations of Mouse Trigeminal Ganglion Neurons Following Orofacial Inflammation Revealed by Single-Cell Analysis
Source: Front Cell Neurosci. 2022 Jun 2;16:885569. doi: 10.3389/fncel.2022.885569 (PMC9200971; doi:10.3389/fncel.2022.885569)

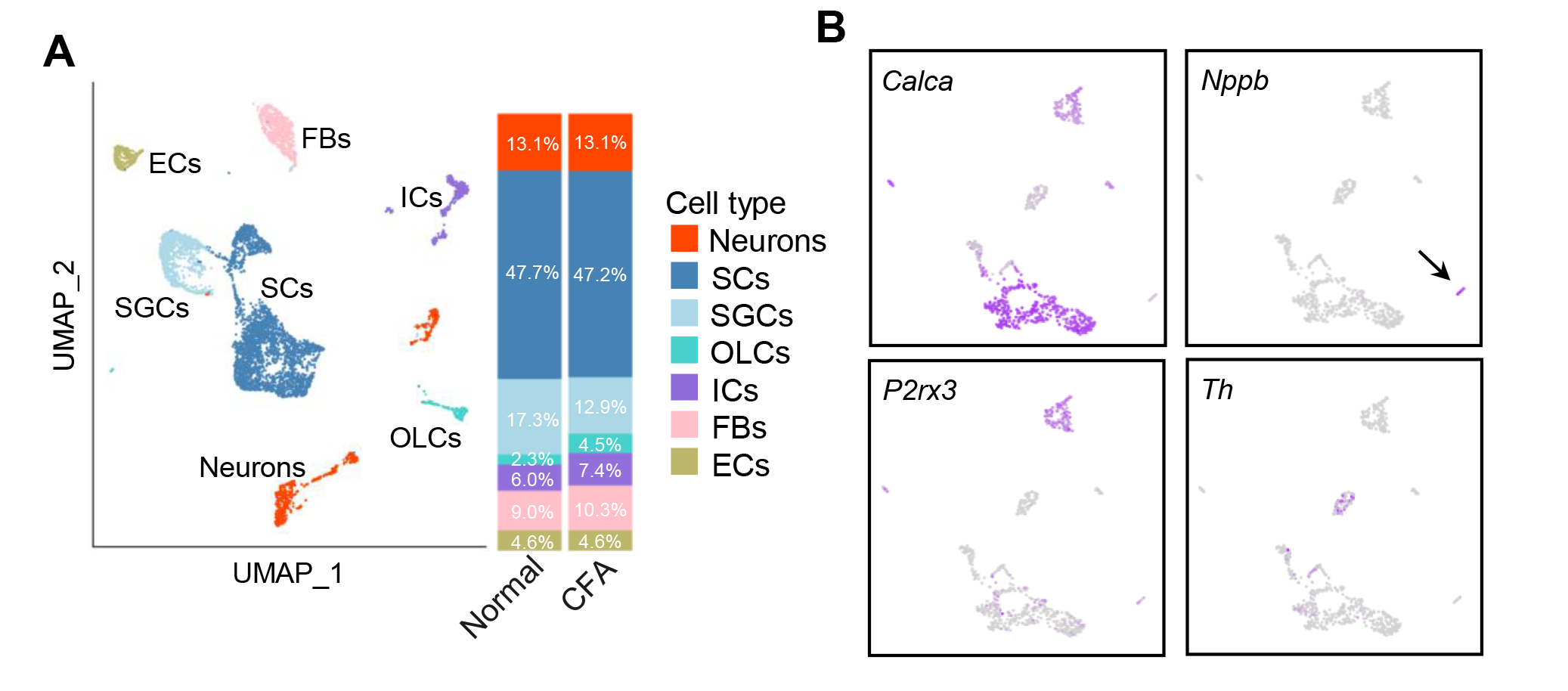

Supplement: Supplementary Figure 1 — (A) UMAP plot of all cells in mouse TGsobtained from normal and experimental groups. The identified seven types of cells in TG. The proportions of each cell type in different groups are displayed in bar plots and [file Image_1.TIF]
